# Disentangling the Relative Importance of Changes in Climate and Land-Use Intensity in Driving Recent Bird Population Trends

**DOI:** 10.1371/journal.pone.0030407

**Published:** 2012-03-30

**Authors:** Sarah M. Eglington, James W. Pearce-Higgins

**Affiliations:** British Trust for Ornithology, The Nunnery, Thetford, Norfolk, United Kingdom; University of Bern, Switzerland

## Abstract

Threats to biodiversity resulting from habitat destruction and deterioration have been documented for many species, whilst climate change is regarded as increasingly impacting upon species' distribution and abundance. However, few studies have disentangled the relative importance of these two drivers in causing recent population declines. We quantify the relative importance of both processes by modelling annual variation in population growth of 18 farmland bird species in the UK as a function of measures of land-use intensity and weather. Modelled together, both had similar explanatory power in accounting for annual fluctuations in population growth. When these models were used to retrodict population trends for each species as a function of annual variation in land-use intensity and weather combined, and separately, retrodictions incorporating land-use intensity were more closely linked to observed population trends than retrodictions based only on weather, and closely matched the UK farmland bird index from 1970 onwards. Despite more stable land-use intensity in recent years, climate change (inferred from weather trends) has not overtaken land-use intensity as the dominant driver of bird populations.

## Introduction

Global biodiversity faces many threats and population declines have been documented across a wide-range of taxa [1], [2]. Habitat destruction and management intensification have been responsible for substantial population declines, range contractions and species' extinctions [3]–[5]. These pressures are projected to worsen during the course of the next century [6] and be exacerbated by increasing effects of anthropogenic climate change [7]. Indeed, there is increasing evidence that recent climatic change is already impacting upon species' distribution and abundance [8]–[10], although few studies have attempted to quantitatively disentangle the relative importance of these two drivers in causing recent population declines [11], [12].

Some 12% of all bird species are classified as threatened, and therefore at risk of extinction over the next 100 years, of which, 85% are at risk from habitat destruction or deterioration [13]. One of the best documented examples of how such changes may drive rapid population declines has been the decline of farmland birds across Europe as a result of land-use and management changes associated with increased agricultural intensification [14]–[16]. However, recent analyses indicate an increasing effect of climate change on common European bird populations and communities, with populations of species associated with warmer conditions having stable or increasing populations, whilst those associated with cool climates are declining [17]–[21].

Whilst the inference from these studies is that climate change has overtaken land-use intensity as a driver of common European bird populations [19]–[21], this has not specifically been tested. Indeed, between-habitat differences in species sensitivity to temperature may confound the detectability of climate change and land-use change impacts using such large-scale analyses [22]. Given the considerable scientific and policy interest in this topic, it is vital to properly attribute recent population changes to these different potential causes, to then inform appropriate policy and management responses. Existing analyses do not however, examine the relative importance of climate change and land-use management change in driving recent population trends; the aim of this paper. To examine this, we use forty-year time-series of national trends in the abundance of farmland birds in the UK to firstly quantify the relative importance of both processes in determining the population growth of 18 farmland bird species that contribute to the composite farmland bird index [16]. Secondly, we use these species-specific models to retrodict recent population trends for each species and infer the relative importance of land-use intensity and climate change (inferred from trends in the weather) in driving long-term population trends from the match between modelled and observed trends.

## Methods

### Bird data

Data from the British Trust for Ornithology's (BTO) Common Bird Census (CBC) and the BTO/Joint Nature Conservation Committee/Royal Society for the Protection of Birds Breeding Bird Survey (BBS) were used to generate annual indices of population change for 18 species making up the UK farmland bird index [23]. The CBC provided data from 1966 until 2000. The BBS survey provided data from 1994 to 2008, with seven-years of overlap between the two surveys in order to enable their combination. Full details of the survey design can be found elsewhere [24], [25]. Despite the switch in methodology, the data from the two surveys can be combined for most farmland birds to produce joint trends of national population size [26] which underpin the UK farmland bird index [23].

In this paper, we used the annual index values from joint UK trends for 18 of the 19 farmland bird species listed by Gregory *et al.* [16], excluding only rook *Corvus frugilegus* because it was recorded on relatively few (<15 per annum) CBC plots, reducing the precision of the early trend. The species included were grey partridge *Perdix perdix,* common kestrel *Falco tinnunculus*, northern lapwing *Vanellus vanellus*, stock dove *Columba oenas*, common wood pigeon *Columba palumbus*, European turtle dove *Streptopelia turtur*, western jackdaw *Corvus monedula*, skylark *Alauda arvensis*, common whitethroat *Sylvia communis*, common starling *Sturnus vulgaris*, Eurasian tree sparrow *Passer montanus*, yellow wagtail *Motacilla flava*, European greenfinch *Carduelis chloris*, European goldfinch *Carduelis carduelis*, common linnet *Carduelis cannabina*, yellowhammer *Emberiza citrinella*, common reed bunting *Emberiza schoeniclus* and corn bunting *Emberiza calandra.* For each species, annual estimates of abundance from 1966–2008 were produced using standard methods [27].

### Land-use data

A wide-range of aspects of farmland management have changed over recent decades (see Wilson *et al* [28] for a review). These can be measured using a wide-variety of metrics, pointing to a general trend towards a loss of semi-natural habitat and increasing intensification during the 1970s and 1980s [14] to produce increased agricultural yields. Whilst some habitat loss may be detectable using remote sensing [29], [30], detecting important changes on farmland associated with altered cropping, the intensity of management, or relatively subtle habitat conversion, such as from wet to dry grassland across large-scales, may be difficult. We therefore follow Donald *et al.* [15] and use the annual yield of wheat and barley in the UK as a measure of arable intensification [31]. Applying the same logic to pastoral systems, we used separate annual estimates of the size of the national cattle herd and sheep flock from June census data [32] as indices of the intensification of livestock-husbandry. Usefully, these measures combine the consequences of changes in both land-cover and land-use intensity.

### Weather data

Temperature data were obtained from the Central England Temperature (HadCET) dataset for a roughly triangular area enclosed by Lancashire, London and Bristol [33]. Rainfall data were taken from the England and Wales Precipitation (EWP) Series [34]. These data therefore match the area from which most of the bird data are derived, particularly for the CBC period. The survival rates of a range of farmland birds are known to correlate with the severity of winter weather [35], whilst breeding success may vary annually in relation to conditions during the breeding season [36]. We therefore used the mean minimum temperature of the coldest month as a measure of winter severity and mean monthly mean temperature and mean monthly precipitation during the bird breeding season (April–July) as measures of annual variation in breeding conditions. For three long-distance migrants, which winter in the semi-arid Sahelian zone south of the Sahara, we replaced minimum temperature with Sahel rainfall, an appropriate measure of weather conditions on the African wintering grounds known to affect survival rates in these species [37]. Total wet season Sahel rainfall (May–October) was derived annually from the 0.5×0.5 degree cell gridded data TS3.0 (http://badc.nerc.ac.uk:80/browse/badc/cru/data), from the Climate Research Unit at UEA via British Atmospheric Data Centre (BADC).

### Statistical analysis

The selection of variables outlined above provided a balanced design of three national land-use time-series to be compared against three national weather time-series, allowing analysis of the impacts of these factors on population growth. The three land-use variables were significantly correlated, meaning that disentangling the relative importance of each of these was difficult, but given the low degree of correlation between the weather variables and land-use variables, this should not affect our assessment of the relative importance of each in driving farmland bird populations (Table 1).

**Table 1:** Pearson correlation coefficients between all climate and land-use variables.

	Min temp	Sahel rain	Breed temp	Breed rain	Cereal yield	Sheep
**Sahel rain**	0.25					
**Breed temp**	0.39	0.21				
**Breed rain**	0.21	0.09	−0.16			
**Cereal yield**	0.18	−0.05	0.48	−0.07		
**Sheep**	0.15	−0.06	0.29	−0.11	**0.78**	
**Cattle**	−0.25	−0.30	**−0.54**	−0.27	**−0.76**	**−0.52**

Pearson correlation coefficients between all climate and land-use variables. Variables in bold indicate correlation coefficients of *r*>0.5. For a description of the variables, refer to methods.

### i Relative importance of weather and land-use in driving population growth

We used hierarchical partitioning (HP) to determine the independent contribution of these explanatory variables to each bird population trend [38]. HP addresses the presence of collinearity by determining the independent contribution of each explanatory variable to the response variable and separates it from the joint contribution, resulting from correlation with other variables [39]. The Hier.part macro [40] was run in SAS v.9.2 [41] on each of the 18 species. We therefore modelled annual variation in population growth (ln(n_t+1_/n_t_)) as a function of the index (n) in year_t_ (to account for density dependence), three weather (W) variables; previous annual minimum temperature or Sahel rainfall (W_t_), previous breeding season temperature (W_b_) and rainfall (W_r_), and three land-use (L) variables from the previous year; cereal yield (L_y_), size of the national cattle herd (L_c_) and sheep flock size (L_s_). Variables were related to the previous year as these were most likely to influence population change through productivity in the previous breeding season and over-winter survival. Terms a and b*_n_* describe the intercept and model coefficients respectively, whilst ε represents residual error.
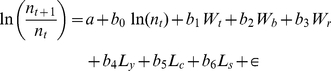
(1)


### ii Relative importance of climate and land-use in driving population trends

We used the models from *i* to retrodict the species-specific population trends from 1966–2008 on the basis of annual variation in weather (which through time describe effects of climate change) and land-use, starting from an initial population value of 1 in 1966, but replacing the observed index (n_t_) with that predicted from the model. The model was therefore free-running from the first year, with annual variation predicted only from variation in the six predictor variables, enabling the ability of each model to track the observed fluctuations in species abundance to be tested. To separate the effects of climate change and land-use intensity, this process was repeated, but fixing the values for either the weather or land-use variables to the mean of the first six years (1965–1970), prior to the major change in agricultural practices in the 1970s [14] or recent climate change [20]. This produced two restricted models where predicted variation in each species trend resulted from either land-use intensity or weather variation only, plus the potential effect of density-dependence.

In order to summarise these models across all species, we replicated the production of the farmland bird indicator, which is a geometric mean of the population trends, but used each of the modelled species trends from ii, instead of the observed trends. Thus, we produced a modelled indicator based on land-use and weather, or the restricted land-use only or weather only models, and compared the fit of these to the real indicator, which runs from 1970.

To test how the relative importance of climate change and land-use intensity has changed through time, the fit (r) of each restricted species model to the observed population trend was assessed for sequential 10-year time-slices through the entire population time-series and averaged across the 18-species. We expect a closer fit between the predictions from the land-use only models and population trends than the weather only models at the start of the time-series and through the period of farmland bird decline [14], but if climate change has now become the dominant driver of farmland bird populations we expect that the performance of the weather-only models will have increased through time and now have greater predictive ability in describing recent population trends than the land-use only models.

## Results

### i Patterns of change in environmental drivers

**Figure 1:**
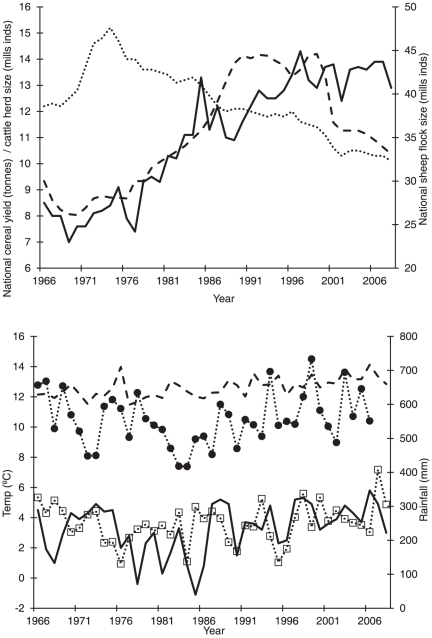
Temporal trends in land-use and weather variables. Temporal trends in a) the total cereal yield (solid line), cattle herd (dotted line) and sheep herd (dashed line) in the UK. Cereal yield (millions tonnes) and cattle herd (millions adults) units given by the left (y)-axis and sheep herd (millions adults) by the right (z)-axis, and b) Mean minimum temperature of the coldest month (solid black line), mean monthly temperature during the breeding season (dashed line), total rainfall during the breeding season (open squares, dotted line), and Sahelian rain (mm/10) (filled circles, dotted line).

There have been significant increases in minimum temperature (r = 0.31, n = 43, *P* = 0.04) and breeding season temperature (r = 0.61, n = 43, *P*<0.0001) from 1966–2008, but not breeding season rainfall (r = 0.07, n = 43, *P* = 0.64) or Sahel rainfall (r = 0.06, n = 43, *P* = 0.71). Strong trends in land-use variables were for increases in cereal yield (r = 0.94, n = 43, *P*<0.0001) and the size of the sheep flock (r = 0.67, n = 43, *P*<0.0001), although with a recent decline post the food-and-mouth disease outbreak in 2001, and reductions in the size of the cattle herd (r = −0.81, n = 43, *P*<0.0001) from 1975. These trends are illustrated in Figure 1.

### ii Relative importance of weather and land-use in driving population growth

**Figure 2:**
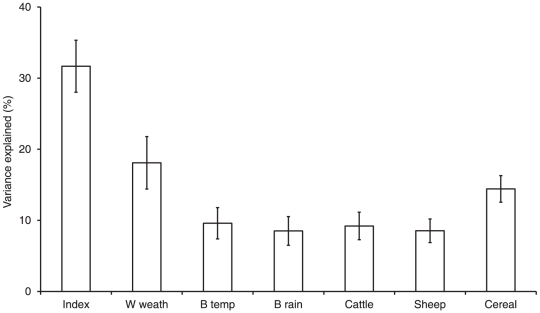
The mean variance (± se) of population growth attributable to each variable. Results of hierarchical partitioning, showing the mean variance (± se) of population growth attributable to each variable across all species. Variances sum to 100%. Log Index t-1 (Index) is included to account for potential density-dependence, winter weather (W weath, combining the effects of minimum temperature for residents and Sahel rainfall for migrants), breeding season temperature (B temp) and breeding season rainfall (B rain) are weather variables, and Cereal, Cattle and Sheep describe land-use intensity.

There was a largely even split in the partitioning of variation in population growth between count in the previous year (32%), weather (36%) and land-use (32%). Thus weather and land-use were approximately equally important in determining annual variation in population growth rates of farmland birds (Figure 2). Given that the models accounted for an average of 40±3% of the variation in population growth, about 14% of the annual variation in farmland bird populations was driven by weather and 13% by land-use change.

### iii Relative importance of climate and land-use in driving population trends

**Figure 3:**
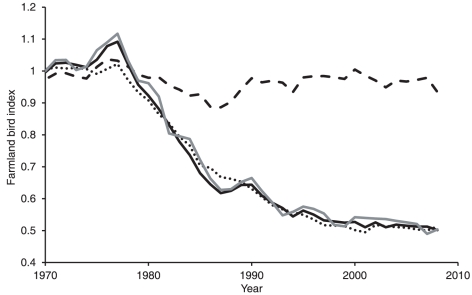
Modelled UK farmland bird indicator based on land-use intensity and weather. Modelled UK farmland bird indicator based on land-use intensity and weather (black solid line), or the restricted land-use intensity only (black dotted line) or weather only (black dashed line) models, compared to the real indicator (grey solid line), which runs from 1970 [25].

**Figure 4:**
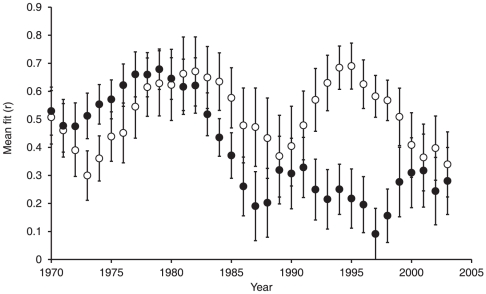
Changes in importance of land-use intensity and climate change in driving farmland bird population growth. Changes in the relative importance of land-use intensity and climate change in driving farmland bird population growth. The graph shows the fit of the land-use (open circles) and weather (black circles) only models to the observed population trend, as assessed from the correlation coefficients between observed and predicted populations for sequential 10-year time-slices, and plotted against the central year.

Models of population growth (Table S1) had good explanatory power in retrodicting observed population trends of farmland birds (Figure S1), with a mean coefficient of determination between observed and expected index values of r^2^ = 0.88±0.03. The mean coefficient of determination of the land-use only models was r^2^ = 0.79±0.06 and the weather only models r^2^ = 0.34±0.08. Population trends of the majority of species were therefore much more closely related to land-use change than climate change, with weather only models producing a better fit to the observed trend than land-use only models in only two species. The importance of land-use intensity in explaining the observed farmland bird decline is indicated by the close-fit of the modelled trend in the indicator when based upon both land-use intensity and weather (r^2^ = 0.99), or just land-use intensity (r^2^ = 0.98). In the absence of land-use change the coefficient of determination was weak at r^2^ = 0.19 with only a 7% population decline predicted, rather than the observed 50% (Figure 3). At the start of the time-series, weather only models provided a better fit to the farmland bird population trends than land-use only models, but this switched in 1981 (reflecting correlations from 1976–1985, the period of major population decline). During the 1990s, observed trends were strongly driven by land-use intensity rather than weather (Figure 4). However, in recent years, the gap has narrowed, although contrary to our predictions, climate change has not exceeded land-use intensity as the main driver of farmland bird population trends. The relative performance of both models is now lower than previously, which reflects the recent stability and reduced variation in farmland bird abundance (Figure 3, Figure S1).

## Discussion

Farmland bird populations have fluctuated as a result of annual variation in both land-use intensity and weather. Whilst a wide-range of previous studies have documented declines in farmland birds as a result of agricultural intensification [e.g.14], [15], and the importance of weather in determining fluctuations of these bird populations [42], this is the first time the relative importance of particular facets of both have been quantified, and related to long-term trends. When modelled together, both were found to have similar explanatory power in accounting for annual changes in population growth. Annual changes in 10 of the 18 species included in this analysis were significantly correlated with at least one of the three land-use variables, of which the main driver appeared to be increasing intensification of arable systems. Annual changes in populations of 9 species were significantly related to weather variables, of which minimum temperature was the most important. Thus, the year to year changes in farmland bird populations observed in the UK may be equally a result of weather or agricultural management during the previous year, which combined with density-dependence, account for 40% of the variation in annual population fluctuations.

Despite the similarity in the annual population fluctuations explained by land-use intensity or weather, modelled population trends incorporating land-use intensity were much more closely linked to observed farmland bird population trends than models based only on weather variables, describing the effects of climate change through time. The importance of land-use intensity results from the strong directional trends in farmland intensification [14], which have exceed the pace of climate change (Figure 1). It is these strong directional trends in land-use intensity which therefore explain why farmland bird populations have declined so precipitously [15], and also suggests that the recent levelling of farmland bird population trends is largely a function of more stable agricultural production, potentially associated with increased uptake of agri-environment schemes aimed at benefiting wildlife [43], although the evidence for beneficial effects of such schemes is as yet equivocal [21]. Whilst it is possible that other, untested factors may also be important, the strength of the relationships identified in our analysis, and the existing literature, suggests we have considered the most important factors which influence farmland bird populations. For example, increasing predator populations, another potential driver of change, have been shown to have little impact on most of the species considered [44].

There is increasing evidence for climate change impacting upon European bird populations, with a general trend towards stable or increasing populations in species associated with warm climates and declines in populations of species associated with cool climates [17], [18]. Our results suggest that for farmland birds, the suite of European bird species most strongly affected by land-use intensity, climate change has been a relatively unimportant driver of long-term trends, even in recent years. We might have expected climate to have become an increasingly important driver of bird populations since the mid-1980s as a result of recent warming [20], [45], but this was not the case, with land-use intensity remaining the dominant driver of farmland bird trends during the 1990s (Figure 3), and no evidence of a recent switch to climate becoming more important. Although annual variation in individual population trends and the farmland bird indicator may be related to weather, such as population increases in the mid-1970s and more rapid declines in the mid-1980s than expected from land-use, our results suggest that apart from one or two exceptions, any impact of climate change on farmland birds has been exceeded by the strong directional shift in land-use intensity. This suggests that the patterns outlined by country- or continent-wide analyses of recent changes in bird communities in response to increases in temperature do not necessarily apply to all environments and have not been reflected in recent changes to the UK farmland bird community. Recent reductions in the community specialisation of birds on farmland [21] may therefore have been largely driven by land-use rather than climate change.

Looking forward, our models suggest that if climate change becomes more severe, we would expect more significant changes to occur, with likely recoveries (or continued recoveries) in corn bunting, European goldfinch, common linnet, common reed bunting and skylark populations as a result of increased survival in milder winters, and increases in grey partridge, yellow wagtail and potentially western jackdaw populations as a result of increased productivity in warmer, drier summers (Table S1). However, given likely further significant shifts in agricultural practices either in response to climate change [46] or other social, political and economic drivers in Europe, the experience of the last forty years of farmland bird monitoring in the UK suggests that it will be changes in land-use intensification which will continue to be the major driver of population change in these species. Given the likely magnitude of future land-use change elsewhere in the globe anticipated as a result of increased requirements for food production and increased effects of climate change [47], understanding the relationship between land-use intensity and bird populations will continue to be a high priority, even during a period of changing climate. On this basis, effects of climate change on biodiversity may therefore be most detectable and important in environments where the level of human exploitation and management is relatively low. Importantly, the fact that the impacts of land-use change can exceed climate change impacts on species, at least in some circumstances, provides evidence that improving land-use practices for bird populations may provide an opportunity to counter negative climate change impacts, and therefore deliver effective climate change adaptation for biodiversity. Testing this further should be a high priority [48].

## Supporting Information

Figure S1 **Modelled population trends of each farmland bird species compared to observed population trends.** Modelled population trends of each farmland bird species compared to observed population trends. Modelled trends were retrodicted from the model of annual variation in population growth (Table S1) applied to the observed index in the first year. Subsequent predicted index values were modelled sequentially from the prediction of population growth applied to the previous predicted index value. Black line = species-specific index of abundance, red line = modelled index (land-use intensity and weather), blue line = restricted model (weather only), green line = restricted model (land-use intensity only). The gap in the index of abundance in 2001 is due a lack of data collected as a result of the food-and-mouth disease outbreak.
(DOC)

Table S1 **Summary of the relationship between predictor variables and population growth for 18 farmland birds.** Summary of the relationship between predictor variables and population growth for 18 farmland birds. For a description of the variables, refer to methods. The number of symbols indicate statistical significance as follows: 1, *P*>0.06; (1), 0.05<*P*<0.06; 2, 0.01<*P*<0.05; 3, 0.001<*P*<0.01; 4, 0.0001<*P*<0.001. The number of significant (*P*<0.05) positive and negative relationships with each predictor variable are given in the last row.
(DOC)
