# Supplementary material for: Disentangling the Relative Importance of Changes in Climate and Land-Use Intensity in Driving Recent Bird Population Trends
Source: PLoS One. 2012 Mar 30;7(3):e30407. doi: 10.1371/journal.pone.0030407 (PMC3316534; doi:10.1371/journal.pone.0030407)
Supplement: Figure S1 — Modelled population trends of each farmland bird species compared to observed population trends. Modelled population trends of each farmland bird species compared to observed population trends. Modelled trends were retrodicted from the model of annual variation in population growth (Table S1) applied to the observed index in the first year. Subsequent predicted index values were modelled sequentially from the prediction of population growth applied to the previous predicted index value. Black line = species-specific index of abundance, red line = modelled index (land-use intensity and weather), blue line = restricted model (weather only), green line = restricted model (land-use intensity only). The gap in the index of abundance in 2001 is due a lack of data collected as a result of the food-and-mouth disease outbreak. (DOC) [file pone.0030407.s001.doc]

a. Corn bunting

b. European goldfinch

c. European greenfinch

d. Grey partridge

e. Western jackdaw

f. Common kestrel

g. Northern lapwing

h. Common linnet

i. Common reed bunting

j. Skylark

k. Common starling

l. Stock dove

m. Eurasian tree sparrow

n. European turtle dove

o. Common whitethroat

p. Common wood pigeon

q. Yellowhammer

r. Yellow wagtail

Figure S1. Modelled population trends of each farmland bird species compared to observed population trends. Modelled trends were retrodicted from the model of annual variation in population growth (Table S1) applied to the observed index in the first year. Subsequent predicted index values were modelled sequentially from the prediction of population growth applied to the previous predicted index value. Black line = species-specific index of abundance, red line = modelled index (land-use intensity and weather), blue line = restricted model (weather only), green line = restricted model (land-use intensity only). The gap in the index of abundance in 2001 is due a lack of data collected as a result of the food-and-mouth disease outbreak.
