# Supplementary material for: Disentangling the Relative Importance of Changes in Climate and Land-Use Intensity in Driving Recent Bird Population Trends
Source: PLoS One. 2012 Mar 30;7(3):e30407. doi: 10.1371/journal.pone.0030407 (PMC3316534; doi:10.1371/journal.pone.0030407)
Supplement: Table S1 — Summary of the relationship between predictor variables and population growth for 18 farmland birds. Summary of the relationship between predictor variables and population growth for 18 farmland birds. For a description of the variables, refer to methods. The number of symbols indicate statistical significance as follows: 1, P>0.06; (1), 0.05<P<0.06; 2, 0.01<P<0.05; 3, 0.001<P<0.01; 4, 0.0001<P<0.001. The number of significant (P<0.05) positive and negative relationships with each predictor variable are given in the last row. (DOC) [file pone.0030407.s002.doc]

Table S1. Summary of the relationship between predictor variables and population growth for 18 farmland birds.

| **Species** | **Log index t-1** | **Min temp** | **Sahel rain** | **Breed temp** | **Breed rain** | **Cattle** | **Sheep** | **Cereal** |
| --- | --- | --- | --- | --- | --- | --- | --- | --- |
| Corn bunting | -- | **+** |  | - | - | + | - | -- |
| European goldfinch | ----- | **++++** |  | (+) | - | + | - | + |
| European greenfinch | - | + |  | (-) | - | - | + | + |
| Grey partridge | -- | - |  | + | -- | + | - | -- |
| Western jackdaw | ----- | - |  | ++ | **++** | **-** | **-** | **+++++** |
| Common kestrel | ----- | + |  | + | - | + | - | (-) |
| Northern lapwing | ---- | (-) |  | + | + | ++ | (-) | - |
| Common linnet | -- | **+++** |  | **+** | **+** | **+** | **+** | **--** |
| Common reed bunting | --- | ++++ |  | - | - | - | (-) | - |
| Skylark | --- | **+++** |  | **-** | **-** | **+** | **-** | **---** |
| Common starling | (-) | + |  | - | - | + | + | - |
| Stock dove | --- | + |  | + | + | + | + | + |
| Eurasian tree sparrow | ---- | + |  | - | - | + | **---** | **----** |
| European turtle dove | - |  | + | -- | - | + | + | - |
| Common whitethroat | ---- |  | +++++ | + | + | + | - | + |
| Common wood pigeon | ----- | - |  | + | + | --- | **+** | ++ |
| Yellow wagtail | ---- |  | + | **-** | **--** | **+** | **+** | **--** |
| Yellowhammer | - | + |  | - | - | + | -- | + |
| **No. sig (-/ +)** | 14 / 0 | 0 / 5 | 0 / 1 | 1 / 1 | 2 / 1 | 1 / 1 | 2 / 0 | 6 / 2 |

Table S1. Summary of the relationship between predictor variables and population growth for 18 farmland birds. For a description of the variables, refer to methods. The number of symbols indicate statistical significance as follows: 1, *P* > 0.06; (1), 0.05 <*P* **<**0.06; 2, 0.01 <*P* **<**0.05; 3, 0.001 <*P* **<**0.01; 4, 0.0001 <*P* <0.001. The number of significant (*P* < 0.05) positive and negative relationships with each predictor variable are given in the last row.
